# Lucid Dreaming and the Feeling of Being Refreshed in the Morning: A Diary Study

**DOI:** 10.3390/clockssleep2010007

**Published:** 2020-02-12

**Authors:** Michael Schredl, Sophie Dyck, Anja Kühnel

**Affiliations:** 1 Central Institute of Mental Health, Medical Faculty Mannheim/Heidelberg University, Zentralinstitut für Seelische Gesundheit, J5, 68159 Mannheim, Germany; 2 Department of Psychology, Medical School Berlin, Calandrellistraße 1-9, 12247 Berlin, Germany

**Keywords:** lucid dreaming, sleep quality, nightmares

## Abstract

REM periods with lucid dreaming show increased brain activation, especially in the prefrontal cortex, compared to REM periods without lucid dreaming and, thus, the question of whether lucid dreaming interferes with the recovery function of sleep arises. Cross-sectional studies found a negative relationship between sleep quality and lucid dreaming frequency, but this relationship was explained by nightmare frequency. The present study included 149 participants keeping a dream diary for five weeks though the course of a lucid dream induction study. The results clearly indicate that there is no negative effect of having a lucid dream on the feeling of being refreshed in the morning compared to nights with the recall of a non-lucid dream; on the contrary, the feeling of being refreshed was higher after a night with a lucid dream. Future studies should be carried out to elicit tiredness and sleepiness during the day using objective and subjective measurement methods.

## 1. Introduction

Lucid dreams are defined as dreams in which the dreamer is aware that he or she is dreaming [1]. In a representative sample, about 50% of the participants reported that they had experienced at least one lucid dream during their lifetime [2]; in student samples, this percentage reaches up to 80% [3]. Its occurrence is associated with a higher perception of self-control, creativity, openness to the experience and having thin boundaries [4,5]. From a physiological point of view, REM periods with lucid dreaming show increased brain activation, especially in the prefrontal cortex, compared to REM periods without lucid dreaming [6,7]. The question arose whether this increased brain activity associated with lucid dreaming might interfere with the process of restoration—one function of sleep—and, thus, reduce sleep quality and/or the feeling of being refreshed in the morning. 

So far, only studies relating inter-individual differences in lucid dream frequency to measures of sleep quality have been carried out. Whereas the study of Denis and Poerio [8] revealed no significant correlation between lucid dreams and subjective sleep quality, two studies [9,10] reported a significant relationship between the three-item lucid dream subscale of the Iowa Sleep Experience Survey and sleep problems. A subsequent study [11] replicated the finding of a negative relationship between lucid dream frequency and sleep quality in two cross-sectional samples. However, this relationship was no longer significant if nightmare frequency was statistically controlled. This indicates that persons with lucid dreams who also have nightmares more often [12] report poorer sleep quality because of the nightmares and not because of their lucid dreams. The association between nightmares and reduced sleep quality is well-established [13,14]. To summarize, the cross-sectional studies looking at the relationship between overall lucid dream frequency and measures of sleep quality indicate that there is no direct relationship between lucid dreaming and non-refreshed sleep and/or poor sleep quality. However, there might be a large number of possible confounding variables, for example, nightmare frequency [11], that can affect the relationship between inter-individual differences in lucid dream frequency and subjective sleep quality. Other candidates might be the openness to experience personality trait that are related to lucid dream frequency [5] and also to nightmare frequency [15] or boundary thinness [16]. In order to overcome this methodological limitation, longitudinal studies comparing subjective sleep parameters of nights with lucid dreams compared to nights without lucid dreams within participants are necessary.

Using unpublished data from a lucid dream induction study, this report aims to investigate the difference in sleep duration and the feeling of being refreshed in the morning after nights where a lucid dream is remembered compared to nights with the recall of a non-lucid dream within participants. As there is no clear theoretical foundation for lucid dreaming affecting the recovery function of sleep, the analyses were exploratory. As the wake-up-back-to-bed technique for induction of lucid dreams involves a deliberate awakening in the early morning and, thus, the disruption of sleep [17], we hypothesized that practicing this technique might interfere with the feeling of being refreshed in the morning.

## 2. Results

Overall, participants in the reality check group and control group recalled at least some dream content in 1413 nights out of 3378 nights (Recall rate: 41.8%). The data presented in Table 1 indicate that sleep in nights with dream recall was significant longer than in nights without dream recall. Similar, the feeling of being refreshed in the morning was higher after nights with dream recall compared to nights without dream recall. If sleep duration was added to the mixed-model analysis, the relationship between dream recall and the feeling of being refreshed was still significant (*F* = 5.0, *p* = 0.026; effect of sleep duration on the feeling of being refreshed: *F* = 1898.9, *p* < 0.0001). 

Lucid dreams in the reality check group and control group were reported after 391 nights, that is, 11.6% of all nights with successful dream recall. Whereas sleep duration did not differ between nights with lucid dream recall and nights without lucid dream recall, the feeling of being refreshed in the morning was slightly higher in the lucid dream condition compared to nights with non-lucid dream recall (see Table 2). If sleep duration was added to the analysis, the difference was still significant (*F* = 6.5, *p* = 0.011), with a considerable effect of sleep duration (*F* = 593.7, *p* < 0.0001). This analysis was repeated for the control group only. The feeling of being refreshed in the morning was marginally more positive after nights with lucid dreams compared to nights with the recall of a non-lucid dream (2.61 ± 0.96 (*N* = 208) vs. 2.45 ± 0.90 (*N* = 533), *F* = 3.7, *p* = 0.055) with a significant effect on the covariate sleep duration (*F* = 402.9, *p* < 0.0001). 

Lastly, the effect of carrying out the wake-up-back-to-bed technique on sleep was studied. Interestingly, total sleep duration was significantly longer in the wake-up-back-to-bed condition (see Table 3), i.e., the participants self-selected nights where they could sleep in. The feeling of being refreshed in the morning did not differ between nights with the wake-up-back-to-bed protocol compared to those without the wake-up-back-to-bed protocol. However, the feeling of being refreshed was significantly lower if sleep duration was statistically controlled: *F* = 6.1, *p* = 0.013 (effect of sleep duration: *F* = 747.5, *p* < 0.0001). Adding a third variable (having a lucid dream or not) in addition to the wake-up-back-to-bed condition (Yes/No) and sleep duration to the model, the feeling of being refreshed was associated positively with having a lucid dream (*F* = 7.1, *p* = 0.008), negatively with doing wake-up-back-to-bed (*F* = 4.4, *p* = 0.036), and positively with sleep duration (*F* = 304.8, *p* < 0.0001).

## 3. Discussion

The diary data analyzed in the present article clearly indicate that there are no negative effects of having a lucid dream on the feeling of being refreshed in the morning; on the contrary, there is a small but significant positive effect of the lucid dream occurrence on the feeling of being refreshed. However, if participants practice a particular method for inducing lucid dreams (the wake-up-back-to-bed procedure) the feeling of being refreshed in the morning is reduced if sleep duration is statistically controlled. 

The major methodological issue related to the interpretation of the data is the selection of the sample. The participants were aware that the study was aiming to induce lucid dreams, that is, that they were interested in the topic. This might result in positive emotions if a lucid dream occurred (“I succeeded.”) and this positive emotion might have affected the feeling of being refreshed in the morning. One possible approach would be to include objective and subjective measures of daytime sleepiness and tiredness, like the maintenance of wakefulness test (MWT), pupillography, vigilance tests, or the Karolinska sleepiness scale (KSS) [18]. Another option to minimize this possible bias would be to carry out a sleep diary study, i.e., focusing primarily on sleep and not on lucid dream induction, and include a few dream-related items at the end. In that case, the participants would not be focusing on lucid dreaming too much. In the present study, subjective estimates of sleep duration were analyzed; it would be very interesting to back up this data with actigraphy data in future studies. A clear advantage of the present study is using the mixed-model approach to analyze intra-individual differences between lucid and non-lucid nights, i.e., inter-individual differences, e.g., chronotype, possible co-morbidities, personality traits, habitual sleep patterns, as this did not affect the results. From a theoretical viewpoint, it would be interesting to include day-to-day measures of stress, evening mood and bedtimes in order to study whether these parameters might affect the occurrence of lucid dreams. 

The statistically significant effect of intra-individual fluctuations of sleep duration on dream recall frequency supports the validity of the present findings, as this relationship (increasing percentage of dream recall with longer sleep duration) has been reported previously [19,20]. Interestingly, there was also a small positive relationship between the feeling of being refreshed in the morning and successful dream recall. As nightmares are related to poor sleep quality [21], one might speculate that positive dream emotions might contribute to this effect.

The findings do not indicate a possible reduction regarding the recovery function of sleep due to the increased brain activity associated with lucid dreaming; on the contrary, the feeling of being refreshed is positively related to the occurrence of a lucid dream—even if sleep duration is statistically controlled. As mentioned above, one might speculate that the feeling of having succeeded in inducing a lucid dream might have biased the estimation regarding the feeling of being refreshed in the morning; however, the positive relation between the feeling of being refreshed in the morning and the occurrence of a lucid dream was also found in the control participants who were not “under pressure” to produce a lucid dream. In order to extent this research, it would be interesting to assess tiredness and sleepiness during the whole day using objective and subjective measures.

The only negative effect on the feeling of being refreshed in the morning we found was related to carrying out the wake-up-back-to-bed protocol, which includes a major sleep disruption [17]. However, even with this protocol, the feeling of being refreshed was not affected if the participants could sleep as long as they chose—the negative effect was only present if the longer sleep duration variable in the wake-up-back-to-bed method was partialled out. If the wake-up-back-to-bed effect is included in the analysis, the occurrence of a lucid dream in this condition is still associated with higher values of the feeling of being refreshed in the morning. The lack of negative effects of lucid dreaming on the feeling of being refreshed in the morning support the notion that it is important to control for possible confounders, like nightmare frequency, in cross-sectional studies looking at lucid dream frequency and subjective sleep parameters [11].

To summarize, the present data set, based on the five-week long diaries of 149 participants, indicated that there are no negative effects of having a lucid dream on the recovery function of sleep. As this is a home-based study, one might speculate if the feeling of being refreshed in the morning after sleep-lab nights with a lucid dream differs from the feeling of being refreshed in the morning after sleep-lab nights with no lucid dream. The “pressure” to produce a lucid dream in the sleep lab might be very high. To follow up this first longitudinal study, it would be interesting to measure tiredness and sleepiness during the day following a night with recall of a lucid dream vs. control nights without a lucid dream.

## 4. Method

### 4.1. Participants

Overall, 193 persons participated in the study, 149 persons completed the study and were included in the analyses. The first group (wake-up-back-to-bed group) included 50 participants (34 women, nine men, gender was missing in seven cases) with a mean age of 21.07 ± 4.57 years. (*N* = 43). The second group practicing reality checks during the day consisted of 45 participants (36 women, two men, gender was missing in seven cases) with a mean age of 20.32 ± 2.00 years. (*N* = 38). The control group (third group) included 52 participants (34 women, nine men, gender was missing in 10 cases) with a mean age of 20.74 ± 1.82 years. (*N* = 42).

### 4.2. Measurement Instruments

Every morning, the participants completed a set of questions. The first question elicited dream recall: “I can recall my dream.” (no, some recall, yes). For the purpose of the present study, the variable was dichotomized: 0 = no recall and 1 = some recall or yes, recall. The next question elicited total sleep time during the last night by subtracting all wake periods in hours and minutes. The next question, “How refreshing was your sleep?”, was presented in a five-point format: 0 = not at all refreshing, 1 = hardly refreshing, 2 = moderately refreshing, 3 = quite refreshing, and 4 = very refreshing. The item measuring lucidity (“I was lucid.”) was dichotomous (yes/no). During the introduction to the study procedures, participants received the following definition [22]: “In a lucid dream, one is aware that one is dreaming during the dream. Thus, it is possible to wake up deliberately, or to influence the action of the dream actively, or to observe the course of the dream passively”. Lastly, the participants of the wake-up-back-to-bed group were asked whether they adhered last night to the wake-up-back-to-bed protocol. 

### 4.3. Procedure

Participants were recruited at the Medical School Berlin; all were psychology bachelors. They received course credits for participation. After all participants were registered, they were randomized (using an online random number generator) into one of the three groups: wake-up-back-to-bed group, reality check group, and control group. The participants received the instructions of their specific group via email. The wake-up-back-to-bed group should perform the technique once a week at a self-selected day. The protocol followed the instruction given by Erlacher [23]: “Set your alarm to six hours after your estimated sleep-onset, get up and work with the remembered dream or a previous recalled dream for at least half an hour, identify possible dream elements that can trigger lucidity if they occur once more. Lastly, go back to bed with the intention to become lucid if one of the triggers occur in the dream”. The reality check group were instructed to perform a reality check (“Am I dreaming or am I awake?”) five to ten times per day. While doing this, the participants were asked to check whether the surroundings and actions follow the physical laws of waking reality. 

Each participant received an email between 5.15 a.m. and 6.00 a.m. as a reminder to complete the morning questionnaire over the five-week study period (days 1 to day 36) via login on the study’s webpage. The participants of the wake-up-back-to-bed group were divided into subgroups connected via ‘WhatsApp’, so they could report when they got up early in the morning and—if comfortable—report the dream experience they worked on. The participants of the reality check group were also put into ‘WhatsApp’ groups, in order to send them five to ten reminders during the day to perform a reality check. 

### 4.4. Statistical Analysis

Statistical analyses were carried out with the SPSS Statistics 25.0 software package for Windows (IBM, Armonk, New York, USA). Mixed-model analyses were carried out to study the relationship between lucid dream occurrence and the feeling of being refreshed and sleep duration. In order to account for inter-individual variability, the random factor (participants) included the intercept. 

### 4.5. Ethics Statement

Ethical review and approval was not required for the study on student participants of online questionnaire studies in accordance with the local legislation and institutional requirements. With participation the participants agree that their data might be published in a way that participants cannot be identified.

## 5. Conclusions

Despite the heightened brain activity during REM sleep with lucid dreaming, the restorative function of sleep, measured as subjective feeling of being refreshed in the morning, was not negatively affected by having a lucid dream. Applying the Wake-up-Back-to-Bed technique (including a period of wakefulness during the early morning hours) can affect the feeling of being refreshed in the morning, but this effect can be counteracted by extending sleep duration.

## Figures and Tables

**Table 1:** Feeling of being refreshed and sleep duration after nights with dream recall compared with nights without dream recall.

Nights	Feeling Refreshed	Sleep Duration
Night with dream recall	2.56 ± 0.93 (*N* = 1413)	7.73 ± 1.45 (*N* = 1411)
Night without dream recall	2.30 ± 1.00 (*N* = 1964)	7.13 ± 1.62 (*N* = 1965)
	Mixed model *F* = 39.4, *p* < 0.0001	Mixed model *F* = 129.5, *p* < 0.0001

**Table 2:** Feeling of being refreshed and sleep duration after nights with lucid dream recall compared with nights without lucid dream recall.

Nights	Feeling Refreshed	Sleep Duration
Night with lucid dream recall	2.64 ± 0.93 (*N* = 391)	7.78 ± 1.58 (*N* = 391)
Night without lucid dream recall	2.53 ± 0.92 (*N* = 1022)	7.71 ± 1.39 (*N* = 1020)
	Mixed model *F* = 7.5, *p* = 0.006	Mixed model *F* = 1.2, *p* = 0.257

**Table 3:** Feeling of being refreshed and sleep duration after nights with wake-up-back-to-bed protocol compared with nights without wake-up-back-to-bed protocol.

Nights	Feeling Refreshed	Sleep Duration
Night with wake-up-back-to-bed protocol	2.48 ± 0.87 (*N* = 256)	7.92 ± 1.19 (*N* = 256)
Night without wake-up-back-to-bed protocol	2.40 ± 0.92 (*N* = 1486)	7.35 ± 1.40 (*N* = 1487)
	Mixed model *F* = 1.7, *p* = 0.158	Mixed model *F* = 38.8, *p* < 0.0001
